# Genome Mining and Screening for Secondary Metabolite Production in the Endophytic Fungus *Dactylonectria alcacerensis* CT-6

**DOI:** 10.3390/microorganisms11040968

**Published:** 2023-04-08

**Authors:** Qianliang Ming, Xiuning Huang, Yimo He, Lingyue Qin, Yu Tang, Yanxia Liu, Yuting Huang, Hongwei Zhang, Peng Li

**Affiliations:** 1 Department of Pharmacognosy, College of Pharmacy, Army Medical University, Chongqing 400038, China; 2 Drug and Instrument Supervision and Inspection Station, 32339 Troops of the Chinese People’s Liberation Army, Lhasa 850015, China

**Keywords:** endophytic fungi, genome sequencing, antiSMASH, biosynthetic gene cluster, secondary metabolite

## Abstract

Endophytic fungi are a treasure trove of natural products with great chemical diversity that is largely unexploited. As an alternative to the traditional bioactivity-guided screening approach, the genome-mining-based approach provides a new methodology for obtaining novel natural products from endophytes. In our study, the whole genome of an endophyte, *Dactylonectria alcacerensis* CT-6, was obtained for the first time. Genomic analysis indicated that *D. alcacerensis* CT-6 has one 61.8 Mb genome with a G+C content of 49.86%. Gene annotation was extensively carried out using various BLAST databases. Genome collinearity analysis revealed that *D. alcacerensis* CT-6 has high homology with three other strains of the *Dactylonectria* genus. AntiSMASH analysis displayed 45 secondary metabolite biosynthetic gene clusters (BGCs) in *D. alcacerensis* CT-6, and most of them were unknown and yet to be unveiled. Furthermore, only six known substances had been isolated from the fermented products of *D. alcacerensis* CT-6, suggesting that a great number of cryptic BGCs in *D. alcacerensis* CT-6 are silent and/or expressed at low levels under conventional conditions. Therefore, our study provides an important basis for further chemical study of *D. alcacerensis* CT-6 using the gene-mining strategy to awaken these cryptic BGCs for the production of bioactive secondary metabolites.

## 1. Introduction

Endophytic fungi commonly refer to a group of fungi that colonize healthy plant tissues inter- and/or intracellularly, without causing apparent disease symptoms in the host plants [1]. It has been widely reported that endophytic fungi have the ability to aid in the defense of their host plants [2]. More importantly, endophytic fungi are able to biosynthesize a variety of novel secondary metabolites, which can have outstanding potential as leading structures for new drug discovery [3]. These metabolites belong to different structural classes, such as alkaloids, terpenoids, steroids, peptides, polyketides, lignans, phenols and lactones [4,5] and have shown different pharmacological activities, such as anticancer [6], antimicrobial [7], antioxidant [8], antidiabetic [9], anti-inflammatory [10], anti-Alzheimer’s disease [11] and immunosuppressive [12]. Therefore, endophytic fungi represent a treasure trove of bioactive and new natural products with great chemical diversity that have largely been unexploited.

Traditionally, the discovery of novel bioactive natural products with potent bioactivities from endophytic fungi occurs through a process known as bioactivity-guided screening, which is termed a “top-down” approach [13]. Unfortunately, this “top-down” approach has suffered from several pitfalls, such as high frequency of rediscovery of known compounds and difficulty in obtaining natural products produced at trace concentrations. As an alternative to the “top-down” approach, the genome-mining-based approach—which utilizes the abundance and availability of genome data, bioinformatics analysis and gene manipulation tools to search for biosynthetic gene clusters (BGCs) biosynthesizing novel natural products [13,14]—represents a new research methodology for obtaining novel natural products from endophytes. The genome-mining-based approach, known as a “bottom-up” approach, is an efficient way of understanding the biosynthesis of different types of natural products and allows the manipulation of biosynthesis pathways for improvement of yield, activation of silent BGCs and heterologous expression of BGCs [15].

During our previous investigation, an endophytic fungus CT-6, isolated from the medicinal plant *Corydalis tomentella* (Papaveraceae), was screened against three kinds of cancer cells to assess its anticancer potential (Figure S1). In this study, we identified strain CT-6 through its ITS region and 5.8S rRNA sequence. In order to better understand strain CT-6 and explore its secondary metabolite biosynthetic potential, whole-genome sequencing and assembly were implemented by next-generation and third-generation sequencing technologies. Subsequently, gene annotation was extensively predicted using various BLAST databases, including non-redundant (NR) protein sequence, Pfam, Swiss-Prot, Clusters of Orthologous Groups (COG), Gene Ontology (GO), Kyoto Encyclopedia of Genes and Genomes (KEGG) as well as Cytochromes P450, Carbohydrate-active enzymes (CAZy), Comprehensive Antibiotic Research Database (CARD) and Pathogen–Host Interactions (PHI) databases. Genome collinearity analysis between strain CT-6 and other strains in the same genus was also carried out. Furthermore, antiSMASH analysis, metabolites separation and structure determination were performed, partially presenting the biosynthetic potential and chemical diversity of strain CT-6, which provided an important basis for further chemical study of the strain CT-6 using the gene-mining strategy to awaken these cryptic BGCs for the production of more bioactive secondary metabolites.

## 2. Materials and Methods

### 2.1. Strain Source and Culture Medium

Strain CT-6 is an endophytic fungus that was isolated and purified from the medicinal plant *C. tomentella*, which was collected in September 2014 from Jinfo Mountain (N 29.10460, E 107.20736), Chongqing, China. The strain was deposited in the China General Microbiological Culture Collection Center (CGMCC) in Beijing, China, with collection number 23290.

In this study, potato dextrose agar (PDA) medium (200 g potato, 20 g d-glucose, 15 g agar, 1000 mL deionized water), potato dextrose broth (PDB) medium (200 g potato, 20 g d-glucose, 1000 mL deionized water) and rice medium (150 g rice, 1.5 g peptone, 150 mL of tap water) were used as media for propagation of the tested fungus [16].

### 2.2. Phylogenetic Analysis

For phylogenetic analysis, strain CT-6 was grown on PDA medium for 5 days at room temperature. The mycelium of strain CT-6, scraped directly from the surface of the agar culture, was used to extract the genomic DNA through the traditional cetyltrimethylammonium bromide (CTAB) method [17]. The universal ITS primers ITS4 (5′-GGA AGT AAA AGT CGT AAG G-3′) and ITS5 (5′-TCC TCC GCT TAT TGA TATG C-3′) were used for amplicon sequencing of the ITS region and the intervening 5.8S rRNA gene region [18]. The polymerase chain reaction (PCR) product was sent to Sangon Biotech (Shanghai, China) Co., Ltd. for sequencing. The ITS sequences of strain CT-6 were deposited in GenBank and matched against the nucleotide database in the National Center of Biotechnology Information (NCBI) to compare the sequence homology with closely related organisms. Then, the sequences from closely related organisms were downloaded to conduct the phylogenetic analysis using the neighbor-joining (NJ) method; *Clonostachys chloroleuca* (ON495792) was used as an outgroup. Bootstrap analysis was carried out using 1000 replications with MEGA 7.0 software [19].

### 2.3. Whole Genome Sequencing

Strain CT-6 was cultivated in PDB medium at 28 °C for 3 days on rotary shakers at 180 rpm. The mycelia were collected by centrifugation followed by genomic DNA extraction using the Wizard^®^ Genomic DNA Purification Kit (Promega, MD, USA) according to the manufacturer’s instructions. The genomic DNA of strain CT-6 was sequenced using the Illumina HiSeq X Ten platform and the PacBio Sequel II platform. For Illumina sequencing, at least 10 μg of genomic DNA was interrupted to about 400 bp fragments with a Covaris M220 Focused Ultrasonicator (Covaris Inc., Woburn, MA, USA) for sequencing library construction. The sequencing library was constructed according to the NEXTflex™ Rapid DNA-Seq Kit (Illumina, San Diego, CA, USA) method and sequenced on the Illumina HiSeq X Ten platform. For PacBio sequencing, an aliquot of 8 μg of genomic DNA was sheared to 10 kb using a Covaris g-TUBE (Covaris, MA, USA) at 6000 RPM for 60 s using an Eppendorf 5424 centrifuge (Eppendorf, NY, USA). DNA fragments were then end-repaired and ligated with SMRTbell sequencing adapters (Pacific Biosciences, Menlo Park, CA, USA) following the manufacturer’s recommendations. Next, an ~10 kb insert library was prepared and sequenced on one SMRT cell using standard methods.

### 2.4. Genome Assembly

All bioinformatics analyses of the data generated from the Illumina and PacBio platforms were performed on using the Majorbio Cloud Platform (https://cloud.majorbio.com, accessed on 12 April 2022), a free online platform of Shanghai Majorbio Bio-pharm Technology Co., Ltd (Shanghai, China). The genome sequence was assembled using both the Illumina reads and PacBio reads. For Illumina sequence data, the raw data, saved as FASTQ files, were obtained by transferring the original image data into sequence data via base calling. High-quality clean data were obtained by removing connectors and filtering low-quality data according to the statistic of quality information. The PacBio reads were assembled into contigs using Canu (version 1.7). Finally, error correction of the PacBio assembly results was performed using the Illumina clean reads.

### 2.5. Gene Annotation

Prediction of coding gene was performed using Maker2 software (version 2.31.9) [20], tRNA-scan-SE software (version 2.0) [20] was used for tRNA prediction and Barrnap software (version 0.8) [21] was used for rRNA prediction. The predicted coding genes in the whole genome of strain CT-6 were annotated through NR, Pfam, Swiss-Prot, COG, GO and KEGG databases using sequence alignment tools such as BLAST software (version 2.3.0), Diamond software (version 0.8.35) and HMMER software (version 3.1b2) [22]. Briefly, each set of query proteins was aligned with the databases, and annotations of best-matched subjects (E-value < 10^−5^) were obtained for gene annotation. Furthermore, Blast2GO software (version 2.5) [23] was used to obtain the GO annotation information and WEGO software (version 2.0) [24] was used to perform the GO functional classification statistics. Sibelia software (version 3.0.6) [25] was employed in genome collinearity analysis between strain CT-6 and *Dactylonectria estremocensis*, strain CT-6 and *D. macrodidyma* and strain CT-6 and *D. torresensis*, respectively.

### 2.6. Additional Annotation

In order to predict and annotate the presence of cytochrome P450-related genes and CAZy-related genes in strain CT-6, Diamond software (version 0.8.35) was used to align the amino acid sequences of the target species with the cytochrome P450 database [26] and the CAZy database [27], respectively (E-value < 10^−5^). Antibiotic-resistance gene prediction and pathogen–host interaction phenotype classification were also performed using Diamond software (version 0.8.35) within the CARD and PHI databases (E-value < 10^−5^) [28].

### 2.7. Secondary Metabolic Gene Cluster Analysis

The secondary metabolite BGCs of strain CT-6 were predicted using antiSMASH (version 6.1.1) (https://fungismash.secondarymetabolites.org/#!/start, accessed on 25 October 2022) and further annotated using BlastP analysis [29].

### 2.8. Fermentation, Extraction, Isolation and Identification of Secondary Metabolite

Strain CT-6 was activated on PDA medium at room temperature for 10 days, and then the activated fungal hyphae were added to a sterilized 250 mL Erlenmeyer flask containing 100 mL PDB medium for 5 days in a shaker (180 rpm) at 28 °C. After that, 10 mL of the PDB fungal culture was inoculated in a sterilized 500 mL Erlenmeyer flask containing sterilized rice medium (150 g rice, 1.5 g peptone, 150 mL of tap water) and cultured at room temperature for 30 days. The fermented products of 30 flasks were extracted exhaustively with methanol (MeOH) followed by decompressing distillation to acquire a brown extract (310 g). The brown extract was then suspended in a 50% methanol–water solution. After degreasing by petroleum ether (PE), the suspended solution was extracted with ethyl acetate (EtOAc) to afford 56.1 g of EtOAc fraction.

The EtOAc fraction was subjected to column chromatography over silica gel using gradient elution with a mixture of PE/EtOAc/MeOH (5:1:0, 3:1:0, 1:1:0, 1:3:0, 1:5:0, 0:5:1, 0:3:1, 0:1:1, 0:1:3, *v*/*v*) to give six fractions (Fr.1-Fr.6), respectively. After solvent evaporation, a colorless block crystal was crystallized from Fr.4, which was further purified by recrystallization to afford a white solid powder (15.8 g, **1**). Fr.3 was subjected to medium-pressure liquid chromatography on reversed-phase silica gel eluted with a mixture of MEOH/H_2_O, and the yield fraction was further purified by Sephadex LH-20 with MeOH to afford pure compounds **2** (29.1 mg) and **3** (43.7 mg). Fr.2 was subjected to medium-pressure liquid chromatography on silica gel eluted with a gradient of petroleum PE-EtOAc (100:0−0:100), and the yield fraction was further purified by Sephadex LH-20 with MeOH to afford pure compounds **4** (105.4 mg) and **6** (57.1 mg). Fr.5 was subjected to medium-pressure liquid chromatography on reversed-phase silica gel eluted with a mixture of MEOH/H_2_O, and the yield fraction was further purified by Sephadex LH-20 with MeOH to afford pure compound **5** (25.6 mg).

The structures of the six pure metabolites were identified by mass spectrometry (MS) and Nuclear Magnetic Resonance Spectroscopy (NMR). ^1^H and ^13^C NMR spectra were recorded with a Bruker Avance III 400 MHz NMR spectrometer at 25 °C. Low-resolution MS spectra were obtained with a Shimadzu LC-MS-2020 triple quadrupole tandem mass spectrometer equipped with an electrospray ionization (ESI) probe operating in positive or negative ionization mode.

## 3. Results and Discussion

### 3.1. Identification of Strain CT-6

**Figure 1:**
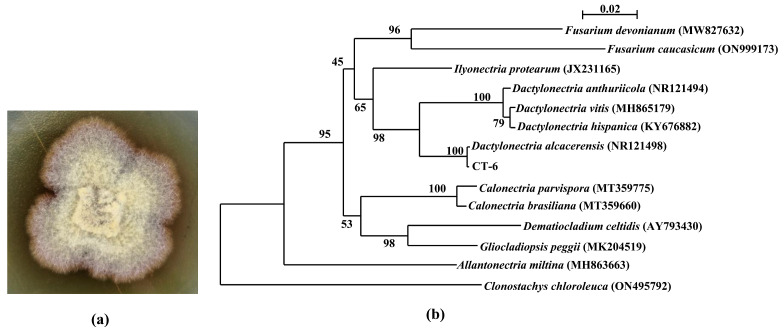
Colony of *D. alcacerensis* CT-6 (**a**) and phylogenetic tree of the endophyte CT-6 based on ITS region and 5.8S sequences (**b**). Bootstrap values greater than or equal to 50% (1000 replicates) are shown at branches.

The hyaline sterile hyphae of strain CT-6 grew from PDA medium after incubation for 4 d at 28 °C, and gradually became yellowish-brown (Figure 1a). Phylogenetic analysis (Figure 1b) displayed that the ITS regions and the intervening 5.8S rRNA sequence of strain CT-6 (GenBank accession No. OP890611) had 100% similarity to the reference strain *Dactylonectria alcacerensis* CBS 129087 (GenBank accession No. NR121498), suggesting that this isolate belongs unambiguously to the species *D. alcacerensis*.

In this study, the endophyte CT-6 was identified as *D. alcacerensis* through ITS region and 5.8S rRNA sequence analyses. It has been reported that species belonging to *Dactylonectria* are phytopathogens causing grapevine root diseases [30], and *D. alcacerensis* has been associated with black foot disease of grapevines in Argentina [31]. Interestingly, in our research, *D. alcacerensis* CT-6 was an endophyte isolated from the medicinal plant *C. tomentella*, suggesting that the fungus which was a phytopathogen in one plant might be an endophyte in another plant.

### 3.2. Genome Sequencing and Assembly

**Figure 2:**
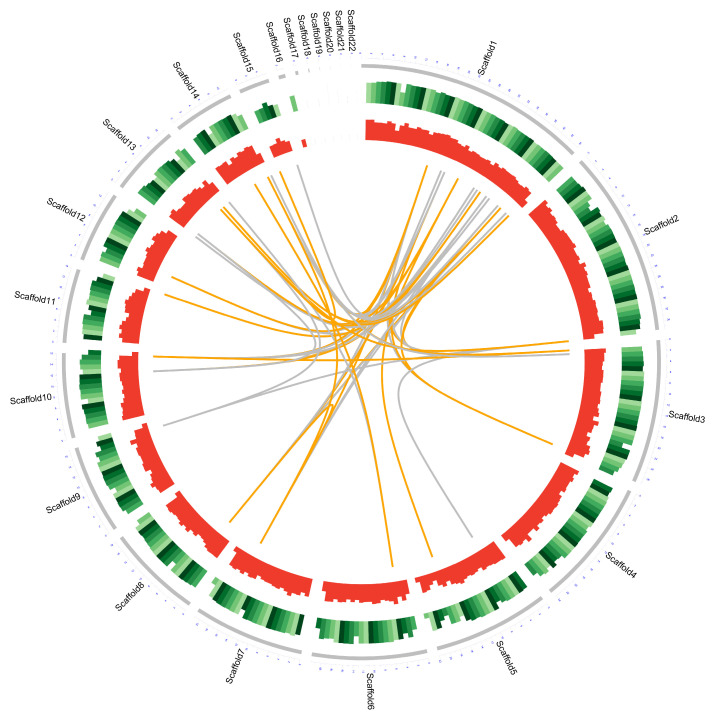
Complete genome map of *D. alcacerensis* CT-6. From outside to inside: scaffolds, GC content (per 200 kb), gene density (per 200 kb) and genome duplication. Regions sharing more than 95% sequence similarity over 5 kb are connected by grey lines. Those with more than 95% similarity over 10 kb are connected by orange lines.

The genome sequence of *D. alcacerensis* CT-6 was assembled and deposited in the NCBI GenBank database (BioProject accession No. PRJNA905207). As shown in Table 1, the genome sequencing of *D. alcacerensis* CT-6 afforded a sequence with a length of 61,760,550 bp, with a maximal length of 8,864,146 bp and a G+C content of 49.86%. The genome consisted of 22 scaffolds with an N50 of 4,367,436 bp and an N90 of 2,702,333 bp. A total of 16,963 protein-coding genes were predicted. The total length of the genes was 36,278,934 bp (accounting for 58.74% of the genome), the average length of these genes was 2138.71 bp and the G+C content in the gene region was 51.30%. For non-coding RNA, we predicted 244 tRNA of 22 types and 67 rRNA (consisting of 56 5S rRNA, 5 5.8S rRNA and 6 28S rRNA). In Figure 2, the genome diagram of *D. alcacerensis* CT-6 shows that there are four circles in the circle diagram, representing (from outside to inside) scaffolds, GC content (per 200 kb), gene density (per 200 kb) and genome duplication.

### 3.3. Genome Annotation

**Figure 3:**
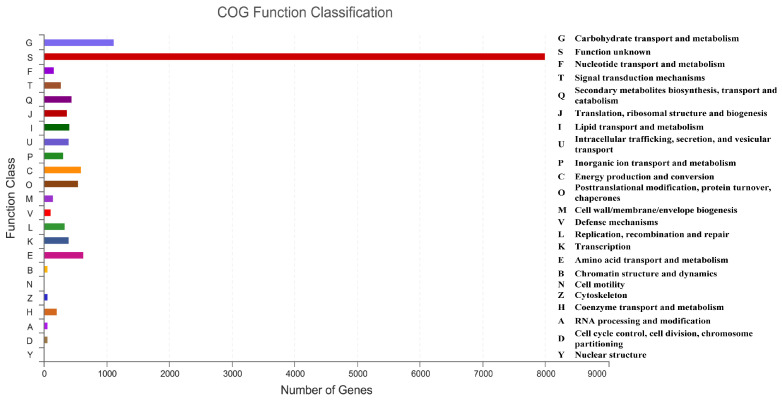
Clusters of Orthologous Groups of proteins (COG) analysis of *D. alcacerensis* CT-6 genes encoding the proteins.

**Figure 4:**
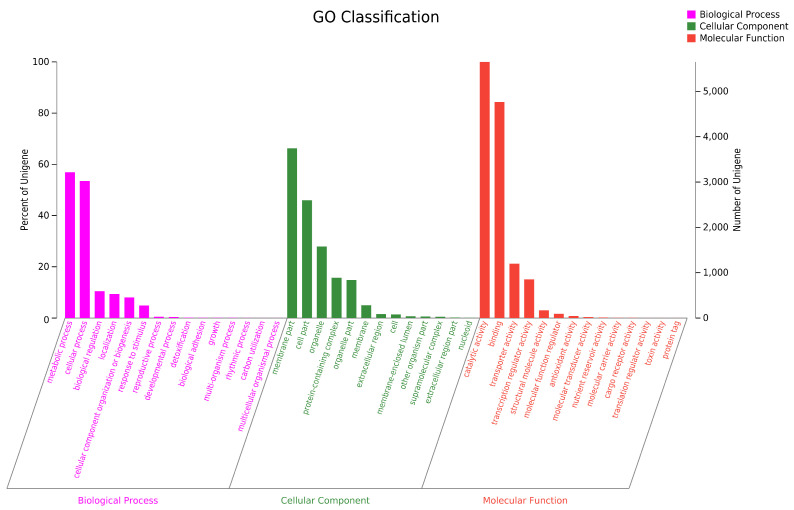
Gene Ontology (GO) analysis of *D. alcacerensis* CT-6 genes encoding the proteins.

**Figure 5:**
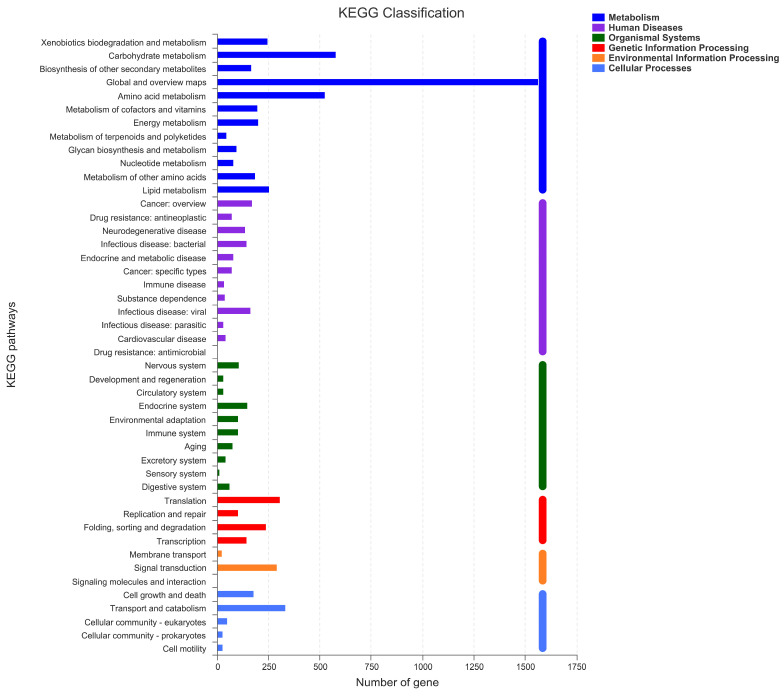
Kyoto Encyclopedia of Genes and Genomes (KEGG) analysis of *D. alcacerensis* CT-6 genes encoding the proteins.

To conduct the functional annotation of the putative-coding sequences in *D. alcacerensis* CT-6, 16,963 non-redundant genes were subjected to a BLAST search function in the NR, Pfam, Swiss-Prot, COG, GO and KEGG databases. A total of 15,558 genes were annotated based on one or more of the six public databases. The largest number of functional genes in *D. alcacerensis* CT-6 was determined as 15,552 genes/91.68% using the NR database, followed by Pfam (11,674 genes/68.82%), Swiss-Prot (10,880 genes/64.14%), COG (14,424 genes/85.03%), GO (10,975 genes/64.70%) and KEGG (4567 genes/26.92%) (Table 1). According to COG analysis, “function unknown” (7990) was associated with the most genes, followed by “carbohydrate transport and metabolism” (1112), “amino acid transport and metabolism” (636) and “energy production and conversion” (595) as the most gene-rich classes in the COG groupings (Figure 3). Based on the GO assignment, 10,975 genes were categorized into 3 main GO categories and 42 subcategories (Figure 4). In terms of biological processes, genes were detected to be involved in metabolic processes (3217) and cellular processes (3021). The cellular component was mainly distributed across the membrane part (3743), cell part (2599) and organelles (1577). Meanwhile, the molecular function revealed that 5650 genes were involved in catalytic activity, followed by binding (4766) and transporter activity (1200). According to KEGG analysis, 4567 genes were annotated and assigned to 46 different KEGG second categories, which could be classified into six main KEGG categories: Metabolism, Human Diseases, Organismal Systems, Genetic Information Processing, Environmental Information Processing and Cellular Processes (Figure 5). “Global and overview maps” (1564) was the most enriched pathway, followed by “carbohydrate metabolism” (580) and “amino acid metabolism” (526).

**Figure 6:**
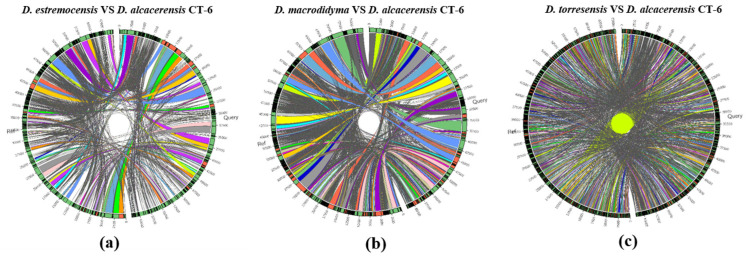
Genome collinearity analysis between *D. alcacerensis* CT-6 and *D. estremocensis* (**a**), *D. alcacerensis* CT-6 and *D. macrodidyma* (**b**), *D. alcacerensis* CT-6 and *D. torresensis* (**c**).

Furthermore, the collinearity relationships between the *D. alcacerensis* CT-6 genome and the reference genome sequences of three other strains of *Dactylonectria* genus whose whole genomes have been sequenced and submitted to the GenBank database (*D. estremocensis*, PRJNA370196; *D. macrodidyma*, PRJNA500112; *D. torresensis*, PRJNA566152) were compared, respectively, using Sibelia (Version 3.0.6) and Circos (Version 0.69-6) software. As shown in Figure 6a–c, the genome collinearity analysis revealed that the *D. alcacerensis* CT-6 genome shows high homology with three reference genomes, and large-scale gene rearrangements were observed between *D. alcacerensis* CT-6 and each of the other three strains of *Dactylonectria* genus.

### 3.4. Additional Annotation

**Figure 7:**
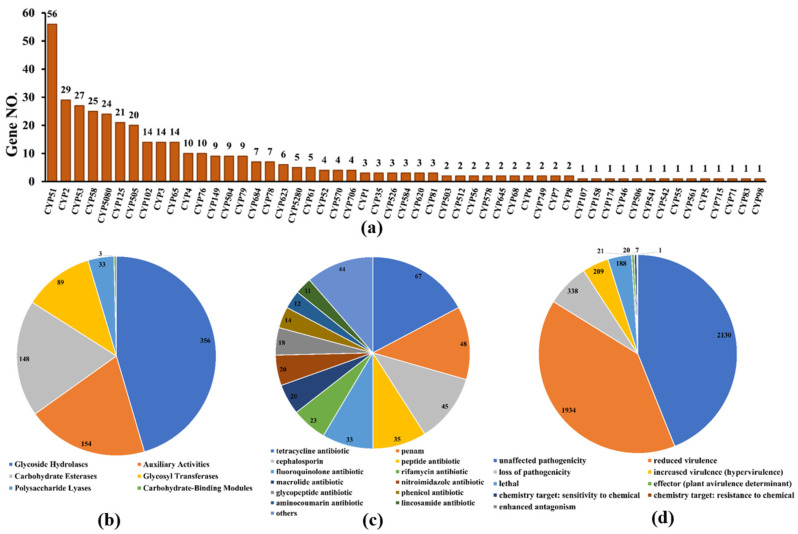
Additional annotation of *D. alcacerensis* CT-6 genes: (**a**) CYP450 annotation; (**b**) CAZy functional classification; (**c**) Antibiotic-resistance gene prediction; (**d**) Pathogen–host interaction phenotype classification.

Cytochrome P450 (CYP450), which is widely distributed in living organisms, is a protein family of mixed functional oxidoreductases with a large superfamily [32]. According to the CYP450 database, 385 putative CYP450 genes consisting of enzymes in 53 superfamilies were identified from the whole genome of *D. alcacerensis* CT-6 (Figure 7a). CYP51 was the superfamily with the most genes (56), followed by CYP2 (29) and CYP53 (27). Furthermore, as one of the most important gene families in the fungal genome, carbohydrate-active enzymes are responsible for the biosynthesis and metabolism of all sugars [33]. Based on the CAZy database assignment, 783 genes were identified as CAZy, including 356 glycoside hydrolases (GHs), 154 auxiliary activities (AA), 148 carbohydrate esterases (CEs), 89 glycosyl transferases (GTs), 33 polysaccharide lyases (PLs) and 3 carbohydrate-binding modules (CBMs) (Figure 7b). 

As far as we know, these organisms, which are prolific producers of antibiotic metabolites, must also be resistant to the antibiotics they produce [34]. Therefore, the analysis of antibiotic-resistant genes in microbial genomes is of great significance to discover what kind of antibiotics are produced by microorganisms. According to the CARD database, a total of 390 genes were annotated as antibiotic-resistant genes from the whole genome of *D. alcacerensis* CT-6 (Figure 7c). “Tetracycline antibiotic” (67) was associated with the most genes, followed by “penam” (48), “cephalosporin” (45) and “peptide antibiotic” (35). Particularly, *D. alcacerensis* CT-6 possesses a large number of antibiotic-resistant genes, suggesting its robust ability to produce different kinds of antibiotics. Based on the PHI analysis, 4373 genes were annotated as PHI genes and classified into nine groups, in which the largest is “unaffected pathogenicity” with 2130 genes, followed by “reduced virulence” (1934), “loss of pathogenicity” (338), “mixed outcome” (209) and “lethal” (118) (Figure 7d).

### 3.5. Analysis of Secondary Metabolite Biosynthetic Gene Clusters

The whole genome sequence of *D. alcacerensis* CT-6 was submitted to the antiSMASH database for secondary metabolite BGCs analysis. AntiSMASH analysis demonstrated that *D. alcacerensis* CT-6 possessed 45 secondary metabolite BGCs, including 15 T1PKS, 8 Terpene, 8 NRPS, 7 NRPS-like, 2 hybrid NRPS+T1PKS, 2 Betalactone, 1 fungal-RiPP, 1 hybrid T1PKS+NRPS-like and 1 hybrid T1PKS+NRPS-like+NRPS biosynthetic genes (Table S1). Most of the BGCs in *D. alcacerensis* CT-6 were unknown and yet to be unveiled. Only 11 of these BGCs (24.44%), which included 6 T1PKS, 2 Terpene, 1 NRPS, 1 hybrid NRPS+T1PKS and 1 hybrid T1PKS+NRPS-like biosynthetic genes, showed similarity to known gene clusters in the MIBiG database (Table 2). Therefore, antiSMASH analysis displayed 45 secondary metabolite BGCs in *D. alcacerensis* CT-6 and only 11 of them showed similarity with known gene clusters, indicating the other 34 BGCs are unknown and yet to be unveiled.

**Figure 8:**
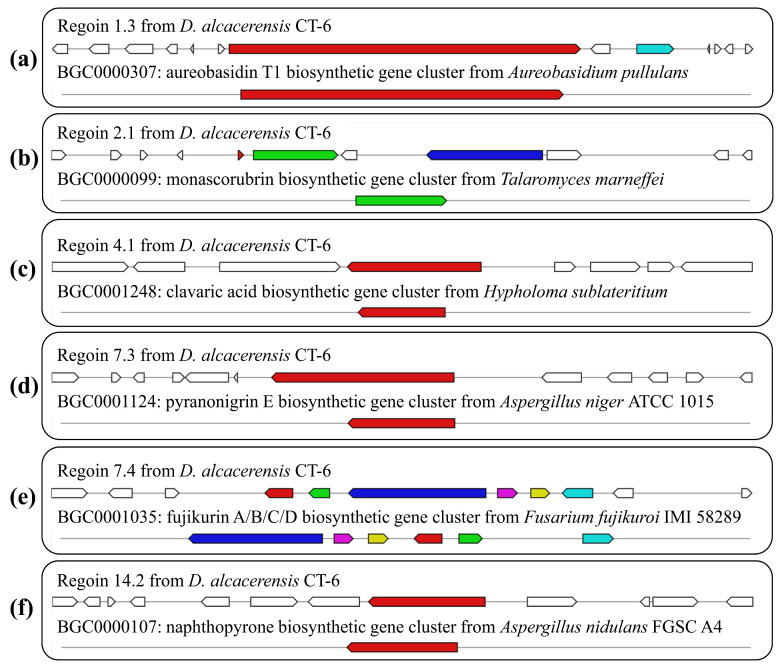
Schematic representation of *D. alcacerensis* CT-6 putative BGCs showing high similarity (100%) with genes from characterized BGCs: aureobasidin T1 (**a**), monascorubrin (**b**), clavaric acid (**c**), pyranonigrin E (**d**), fujikurin A/B/C/D (**e**) and naphthopyrone (**f**). The upper part represents the BGC in *D. alcacerensis* CT-6, followed by the known BGCs in the MIBiG database.

By further comparison, six BGCs with 100% similarity to the gene sequences of other reference strains were identified and predicted to be responsible for the biosynthesis of aureobasidin T1, monascorubrin, clavaric acid, pyranonigrin E, fujikurin A/B/C/D and naphthopyrone (Table 2, Figure 8), respectively. In region 1.3, one NRPS BGC, displaying 100% similarity to the BGC from *Aureobasidium pullulans* (GenBank: EU886741), was responsible for the biosynthesis of aureobasidin T1 (Figure 8a), which is a potent antifungal cyclic depsipeptide with little apparent toxicity in animals [35]. Region 2.1, displaying 100% similarity to the BGC from *Talaromyces marneffei* (GenBank: HM070047), was responsible for the biosynthesis of monascorubrin (Figure 8b), which has been used as a natural red colorant for a wide range of food for more than 1000 years in Asian countries [36]. Region 4.1, displaying 100% similarity to the BGC from *Hypholoma sublateritium* (GenBank: EU665687), was responsible for the biosynthesis of clavaric acid (Figure 8c), which is an antitumor triterpenoid compound and can inhibit the human ras-farnesyl transferase [37,38]. Region 7.3, displaying 100% similarity to the BGC from *Aspergillus niger* ATCC 1015 (GenBank: ACJE01000019.1), was responsible for the biosynthesis of pyranonigrin E (Figure 8d) [39], which is of considerable interest as a potent antioxidant [40]. Region 7.4, displaying 100% similarity to the BGC from *Fusarium fujikuroi* IMI 58289 (GenBank: HF679030), was responsible for the biosynthesis of fujikurin A, fujikurin B, fujikurin C and fujikurin D (Figure 8e) [41], which can play important roles in host–pathogen interactions in several pathogens [42]. Region 14.2, displaying 100% similarity to the BGC from *Aspergillus nidulans* FGSC A4 (GenBank: BN001302.1), was responsible for the biosynthesis of naphthopyrone (Figure 8f) [43].

The other five BGCs showed low similarity (<50%) to the gene sequences of the reference strains (Table 2, Figure S2). Region 1.1, displaying 28% similarity to the BGC from *Talaromyces stipitatus* ATCC 10500 (GenBank: EQ962653), was responsible for the biosynthesis of duclauxin [44]. Region 3.2, which displayed 40% similarity to the BGC from *Aspergillus* sp. Z5 (GenBank: LDZW01000177.1), was responsible for the biosynthesis of squalestatin S1 [45]. Both region 8.2 and region 12.4, displaying 20% similarity to the BGC from *Penicillium brefeldianum* (GenBank: KJ728786.1), were responsible for the biosynthesis of brefeldin A [46]. Region 11.2, displaying 33% similarity to the BGC from *Alternaria oxytropis* (GenBank: KY365741.1), was responsible for the biosynthesis of swainsonine [47].

### 3.6. Secondary Metabolite Isolation and Characterization

**Figure 9:**
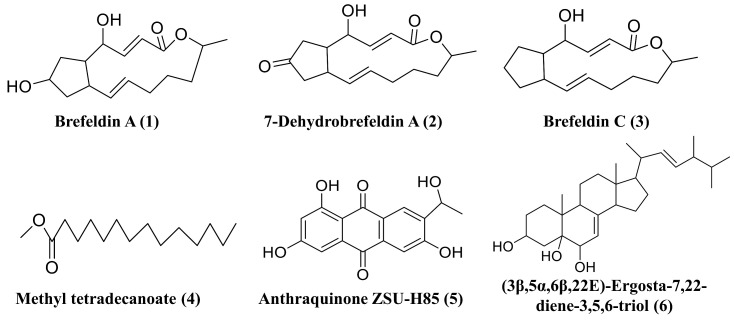
Six secondary metabolites isolated from *D. alcacerensis* CT-6.

To investigate the biosynthetic capacities of *D. alcacerensis* CT-6, six known substances were isolated from the EtOAc fraction of *D. alcacerensis* CT-6. By comparing the MS spectra and NMR data (Figures S3–S8) with those reported in the literature, these metabolites were identified as brefeldin A (**1**) [48], 7-dehydrobrefeldin A (**2**) [49], brefeldin C (**3**) [50], methyl tetradecanoate (**4**) [51], anthraquinone ZSU-H85 (**5**) [52] and (3β,5α,6β,22E)-ergosta-7,22-diene-3,5,6-triol (**6**) [53] (Figure 9).

Among the six known substances, brefeldin A (**1**), 7-dehydrobrefeldin A (**2**) and brefeldin C (**3**) feature a unique cyclopentane skeleton with a 13-membered macrolactone ring that possesses two trans-alkene moieties [54]. Therefore, these three compounds should be biosynthesized by the same BGC. In this study, we isolated a large amount of brefeldin A (**1**) (15.8 g) and small amounts of 7-dehydrobrefeldin A (**2**) (29.1 mg) and brefeldin C (**3**) (43.7 mg). Based on the antiSMASH analysis, two T1PKS BGCs, presumably for brefeldin A (**1**), 7-dehydrobrefeldin A (**2**) and brefeldin C (**3**) synthesis, were found in region 8.2 and region 12.4. However, the two BGCs demonstrated a low degree of amino acid sequence similarity (20%) with the counterpart of *Penicillium brefeldianum* [46], suggesting that the biosynthesis of brefeldin A and its analogs in *D. alcacerensis* CT-6 is though a different BGC from that in *Penicillium brefeldianum*. Brefeldin A was initially known to play a regulatory role in the intracellular transport system and had been found to cause growth inhibition and induce apoptosis and autophagy in several human cancer cells, including leukemia, colon and prostate cancer cells [55,56,57]. Therefore, it was confirmed that brefeldin A is the main effective component of broad-spectrum anti-tumor cell activity in *D. alcacerensis* CT-6. Further investigation of the BGC of brefeldin A biosynthesis is of great significance for the utilization of brefeldin A in drug development. Based on the antiSMASH analysis, methyl tetradecanoate (**4**) and anthraquinone ZSU-H85 (**5**) are putatively biosynthesized by T1PKS BGCs, while (3β,5α,6β,22E)-ergosta-7,22-diene-3,5,6-triol (**6**) is a plausible product of terpene BGC.

Among the secondary metabolites isolated from *D. alcacerensis* CT-6, brefeldin A (**1**) was the predominant product, with relatively abundant yields. Attempts to isolate the products biosynthesized by the BGCs with 100% similarity to that of other reference strains led to failure, probably due to less product accumulation, limitation of separation means or because the BGCs were silent or expressed at low levels under conventional conditions. It should be noted that cultivation under laboratory conditions may not provide all the environmental stimuli required for all the BGCs to produce the corresponding secondary metabolites. Therefore, in order to obtain more bioactive secondary metabolites from *D. alcacerensis* CT-6, further investigation is necessary to understand the expression and regulation mechanism of these BGCs in *D. alcacerensis* CT-6.

## 4. Conclusions

In this study, we assembled and annotated the first high-quality whole genome of the endophytic *D. alcacerensis* CT-6. Although the genome of *D. alcacerensis* CT-6 had high homology with three other strains of the *Dactylonectria* genus, large-scale gene rearrangements were also observed. AntiSMASH analysis displayed 45 secondary metabolite BGCs in *D. alcacerensis* CT-6, and most of them were unknown and yet to be unveiled. Only six known substances have been isolated from the fermented products of *D. alcacerensis* CT-6. Therefore, a great number of cryptic BGCs in *D. alcacerensis* CT-6 are silent and/or expressed at low levels under conventional conditions. Our study provides an important basis for the further chemical study of *D. alcacerensis* CT-6 using a gene-mining strategy to unveil these cryptic BGCs for the production of more bioactive secondary metabolites through various approaches, including induction of mutations, gene knockout, regulation of promoters, transcriptional regulation and heterologous gene expression.

## Figures and Tables

**Table 1:** Genomic assembly and functional annotation of *D. alcacerensis* CT-6.

Item	Value	Item	Value
Total length (bp)	61,760,550	Scaf N90 (bp)	2,702,333
Max length (bp)	8,864,146	All annotation gene	15,558
GC content (%)	49.86	NR	15,552
Gene number	16,963	Pfam	11,674
Gene total length (bp)	36,278,934	Swiss-Prot	10,880
Gene average length (bp)	2138.71	COG	14,424
GC content in gene region (%)	51.30	GO	10,975
Gene/Genome (%)	58.74	KEGG	4567
Scaf No.	22	tRNA	244
Scaf N50 (bp)	4,367,436	rRNA	67

**Table 2:** Putative biosynthetic gene clusters (BGCs) of *D. alcacerensis* CT-6 showed similarity to known gene clusters in the MIBiG database.

Region	Type	From	To	Most Similar Known Cluster (Similarity)
Region 1.1	T1PKS	4,469,169	4,516,076	Duclauxin (28%)
Region 2.1	T1PKS	58,398	124,467	Monascorubrin (100%)
Region 7.4	T1PKS	4,089,485	4,137,285	Fujikurin A/B/C/D (100%)
Region 8.2	T1PKS	220,720	268,510	4-epi-15-epi-brefeldin A (20%)
Region 12.4	T1PKS	1,993,202	2,041,219	4-epi-15-epi-brefeldin A (20%)
Region 14.2	T1PKS	473,269	520,315	Naphthopyrone (100%)
Region 3.2	Terpene	1,283,736	1,305,292	Squalestatin S1 (40%)
Region 4.1	Terpene	2,068,699	2,093,047	Clavaric acid (100%)
Region 1.3	NRPS	6,433,361	6,511,338	Aureobasidin T1 (100%)
Region 7.3	NRPS, T1PKS	2,260,250	2,312,241	Pyranonigrin E (100%)
Region 11.2	T1PKS, NRPS-like	354,933	402,417	Swainsonine (33%)

## Data Availability

Data not included within the manuscript are available upon written request to the corresponding author.

## Supplementary Materials

The following supporting information can be downloaded at: https://www.mdpi.com/article/10.3390/microorganisms11040968/s1. Table S1: Putative biosynthetic gene clusters (BGCs) coding for secondary metabolites in *Dactylonectria alcacerensis* CT-6. Figure S1: The anticancer cell activities of the extraction from the endophyte CT-6 against three kinds of cancer cells: (a) human breast cancer cell line MDA-MB-231 cells, (b) human lung carcinoma cell line A549 cells, (c) human hepatocellular carcinoma cell line SMMC7721 cells. Figure S2: Schematic representation of *D. alcacerensis* CT-6 putative BGCs showing low similarity (<50%) to genes from characterized BGCs. The upper part represents the BGC in *D. alcacerensis* CT-6, followed by the known BGCs in the MIBiG database. Figure S3: The MS and NMR spectra of brefeldin A (**1**): (a) MS spectrum, (b) ^1^H NMR spectrum (CD_3_OD), (c) ^13^C NMR spectrum (CD_3_OD). Figure S4: The MS and NMR spectra of 7-dehydrobrefeldin A (**2**): (a) MS spectrum, (b) ^1^H NMR spectrum (CD_3_OD), (c) ^13^C NMR spectrum (CD_3_OD). Figure S5: The MS and NMR spectra of brefeldin C (**3**): (a) MS spectrum, (b) ^1^H NMR spectrum (CD_3_OD), (c) ^13^C NMR spectrum (CD_3_OD). Figure S6: The MS and NMR spectra of methyl tetradecanoate (**4**): (a) MS spectrum, (b) ^1^H NMR spectrum (CD_3_OD), (c) ^13^C NMR spectrum (CD_3_OD). Figure S7: The MS and NMR spectra of anthraquinone ZSU-H85 (**5**): (a) MS spectrum, (b) ^1^H NMR spectrum (CD_3_OD), (c) ^13^C NMR spectrum (CD_3_OD). Figure S8: The MS and NMR spectra of (3β,5α,6β,22E)-ergosta-7,22-diene-3,5,6-triol (**6**): (a) MS spectrum, (b) ^1^H NMR spectrum (DMSO-d_6_), (c) ^13^C NMR spectrum (DMSO-*d*_6_).
