# Supplementary material for: Genome Mining and Screening for Secondary Metabolite Production in the Endophytic Fungus Dactylonectria alcacerensis CT-6
Source: Microorganisms. 2023 Apr 8;11(4):968. doi: 10.3390/microorganisms11040968 (PMC10142127; doi:10.3390/microorganisms11040968)
Supplement: Supplementary file 1 [file microorganisms-11-00968-s001.zip › microorganisms-2324252-supplementary.pdf]

Microorganisms

Supplementary materials

Genome Mining and Screening for Secondary Metabolite Production in the Endophytic Fungus *Dactylonectria alcacerensis* CT-6

Qianliang Ming [1,2], Xiuning Huang [1], Yimo He [1], Lingyue Qin [1], Yu Tang [1], Yanxia Liu [1], Yuting Huang [1], Hongwei Zhang [2,*] and Peng Li [1,*]

1 Department of Pharmacognosy, College of Pharmacy, Army Medical University, Chongqing 400038, China
2 Drug and Instrument Supervision and Inspection Station, 32339 Troops of the Chinese People's Liberation Army, Lhasa 850015, China
* Correspondence: xuefenghanliu@163.com (H.Z.); pengli@tmmu.edu.cn (P.L.); Tel.: +86-08916731098 (H.Z.); +86-02368771638 (P.L.)

Table S1: Putative biosynthetic gene clusters (BGCs) coding for secondary metabolites in *Dactylonectria alcacerensis* CT-6.

Figure S1: The anticancer cells activities of the extraction from the endophyte CT-6 against three kinds of cancer cells: (a) human breast cancer cell line MDA-MB-231 cells, (b) human lung carcinoma cell line A549 cells, (c) human hepatocellular carcinoma cell line SMMC7721 cells.

Figure S2: Schematic representation of *D. alcacerensis* CT-6 putative BGCs showing low similarity (<50%) with genes from characterized BGCs. The upper part represents the BGC in *D. alcacerensis* CT-6, followed by the known BGCs in the MIBiG database.

Figure S3: The MS and NMR spectra of brefeldin A (1): (a) MS spectrum, (b) ^{1}H NMR spectrum (CD_3OD), (c) ^{13}C NMR spectrum (CD_3OD).

Figure S4: The MS and NMR spectra of 7-dehydrobrefeldin A (2): (a) MS spectrum, (b) ^{1}H NMR spectrum (CD_3OD), (c) ^{13}C NMR spectrum (CD_3OD).

Figure S5: The MS and NMR spectra of brefeldin C (3): (a) MS spectrum, (b) ^{1}H NMR spectrum (CD_3OD), (c) ^{13}C NMR spectrum (CD_3OD).

Figure S6: The MS and NMR spectra of methyl tetradecanoate (4): (a) MS spectrum, (b) ^{1}H NMR spectrum (CD_3OD), (c) ^{13}C NMR spectrum (CD_3OD).

Figure S7: The MS and NMR spectra of anthraquinone ZSU-H85 (5): (a) MS spectrum, (b) ^{1}H NMR spectrum (CD_3OD), (c) ^{13}C NMR spectrum (CD_3OD).

Figure S8: The MS and NMR spectra of (3β,5α,6β,22E)-ergosta-7,22-diene-3,5,6-triol (6): (a) MS spectrum, (b) ^{1}H NMR spectrum (DMSO-d_4), (c) ^{13}C NMR spectrum (DMSO-d_4).

Table S1: Putative biosynthetic gene clusters (BGCs) coding for secondary metabolites in *Dactylonectria alcacerensis* CT-6

No.	Region	Type	From	To	Most similar known cluster (Similarity)
1	Region 1.1	T1PKS	4,469,169	4,516,076	Duclauxin (28%)
2	Region 2.1	T1PKS	58,398	124,467	Monascorubrin (100%)
3	Region 5.3	T1PKS	1,172,788	1,220,783	Unknown
4	Region 6.1	T1PKS	1,384,244	1,432,173	Unknown
5	Region 7.4	T1PKS	4,089,485	4,137,285	Fujikurin A/B/C/D (100%)
6	Region 8.1	T1PKS	12,380	40,112	Unknown
7	Region 8.2	T1PKS	220,720	268,510	4-epi-15-epi-brefeldin A (20%)
8	Region 11.1	T1PKS	214,217	253,794	Unknown
9	Region 12.1	T1PKS	363,327	411,747	Unknown
10	Region 12.3	T1PKS	1,178,521	1,221,759	Unknown
11	Region 12.4	T1PKS	1,993,202	2,041,219	4-epi-15-epi-brefeldin A (20%)
12	Region 13.3	T1PKS	583,252	624,457	Unknown
13	Region 13.4	T1PKS	1,459,028	1,511,477	Unknown
14	Region 14.1	T1PKS	389,886	434,742	Unknown
15	Region 14.2	T1PKS	473,269	520,315	Naphthopyrone (100%)
16	Region 2.2	Terpene	3,736,708	3,757,825	Unknown
17	Region 3.1	Terpene	1,084,549	1,105,303	Unknown
18	Region 3.2	Terpene	1,283,736	1,305,292	Squalestatin S1 (40%)
19	Region 4.1	Terpene	2,068,699	2,093,047	Clavaric acid (100%)
20	Region 5.4	Terpene	2,705,381	2,728,498	Unknown
21	Region 7.2	Terpene	1,569,823	1,590,989	Unknown
22	Region 9.2	Terpene	2,861,857	2,886,598	Unknown
23	Region 13.2	Terpene	338,490	359,698	Unknown
24	Region 1.3	NRPS	6,433,361	6,511,338	Aureobasidin T1 (100%)
25	Region 3.4	NRPS	4,706,754	4,778,695	Unknown
26	Region 4.4	NRPS	4,686,617	4,740,425	Unknown
27	Region 5.1	NRPS	942,389	984,811	Unknown
28	Region 7.1	NRPS	450,669	490,969	Unknown
29	Region 9.1	NRPS	1,130,991	1,184,913	Unknown
30	Region 11.4	NRPS	2,524,119	2,599,155	Unknown
31	Region 12.2	NRPS	666,735	710,651	Unknown
32	Region 1.2	NRPS-like	5,423,605	5,466,610	Unknown
33	Region 2.3	NRPS-like	6,530,723	6,574,674	Unknown
34	Region 3.3	NRPS-like	3,260,777	3,302,241	Unknown
35	Region 3.5	NRPS-like	5,066,642	5,110,579	Unknown
36	Region 4.3	NRPS-like	4,070,003	4,113,999	Unknown
37	Region 11.3	NRPS-like	975,801	1,018,603	Unknown
38	Region 14.3	NRPS-like	1,263,158	1,306,510	Unknown
39	Region 4.2	Betalactone	3,748,476	3,780,599	Unknown
40	Region 13.1	Betalactone	260,919	292,207	Unknown
41	Region 5.2	NRPS, T1PKS	1,027,461	1,079,877	Unknown
42	Region 7.3	NRPS, T1PKS	2,260,250	2,312,241	Pyranonigrin E (100%)
43	Region 10.1	fungal-RiPP	2,068,045	2,103,107	Unknown
44	Region 11.2	T1PKS, NRPS-like	354,933	402,417	Swainsonine (33%)
45	Region 15.1	T1PKS, NRPS-like, NRPS	391,234	453,841	Unknown

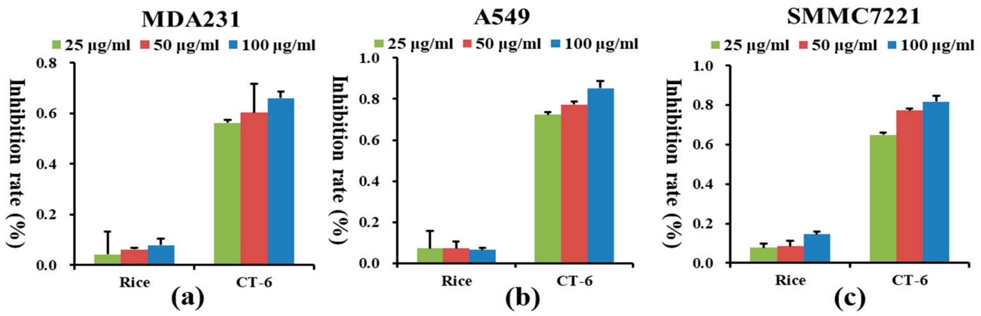

Figure S1: The anticancer cells activities of the extraction from the endophyte CT-6 against three kinds of cancer cells: (a) human breast cancer cell line MDA-MB-231 cells, (b) human lung carcinoma cell line A549 cells, (c) human hepatocellular carcinoma cell line SMMC7721 cells.

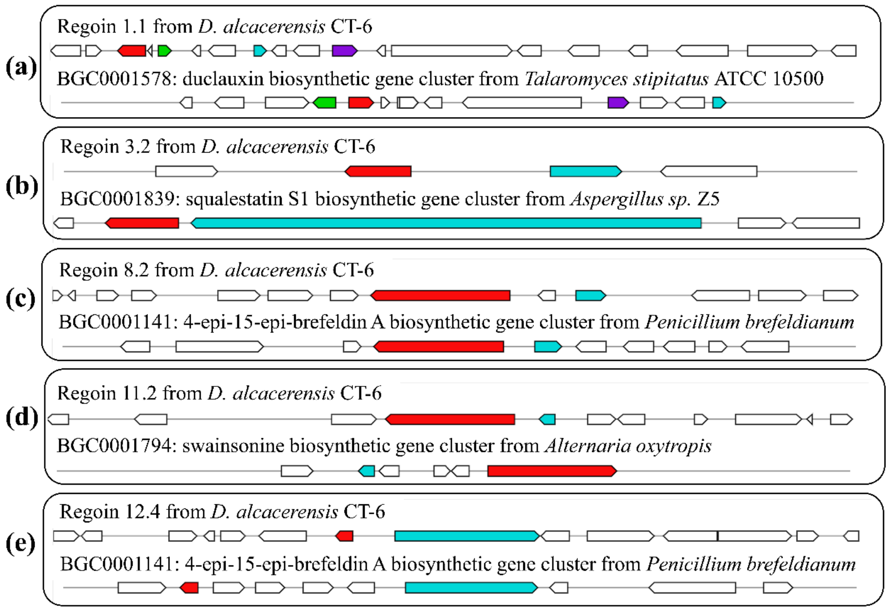

Figure S2: Schematic representation of *D. alcacerensis* CT-6 putative BGCs showing low similarity (<50%) with genes from characterized BGCs. The upper part represents the BGC in *D. alcacerensis* CT-6, followed by the known BGCs in the MIBiG database.

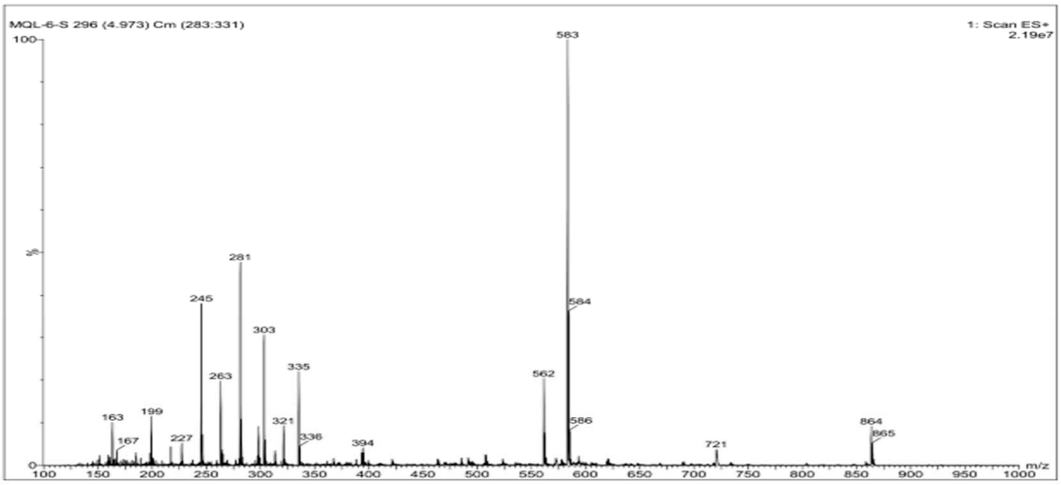

(a)

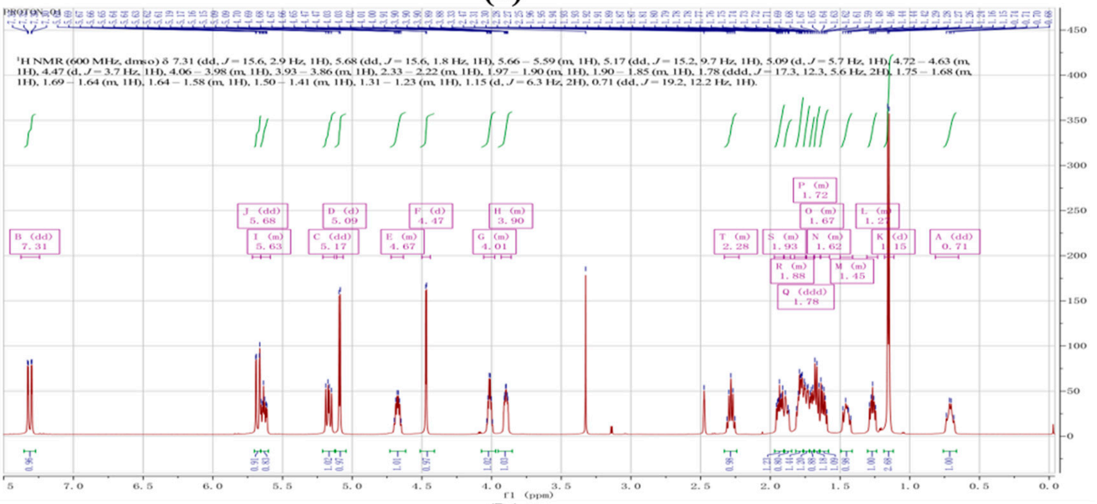

(b)

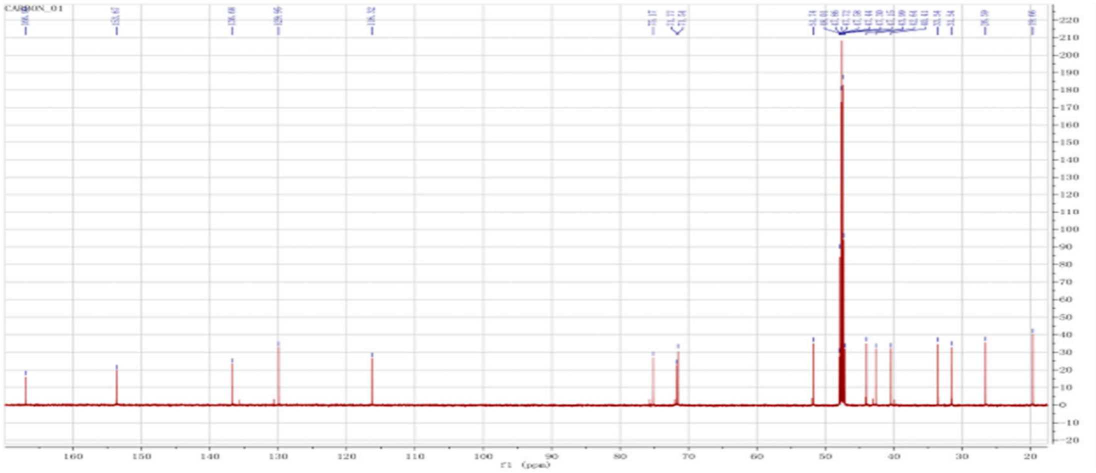

(c)

Figure S3: The MS and NMR spectra of brefeldin A (1): (a) MS spectrum, (b) ^{1}H NMR spectrum (CD_3OD), (c) ^{13}C NMR spectrum (CD_3OD).

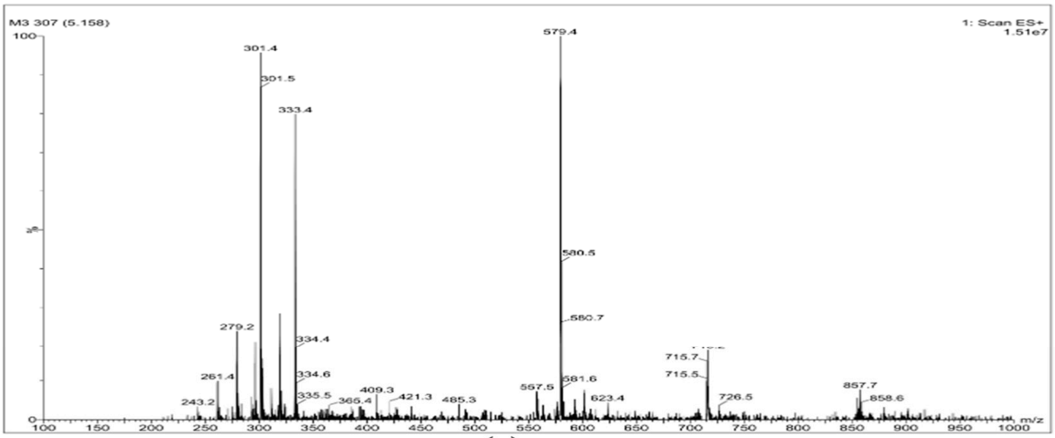

(a)

^{1}H NMR (600 MHz, cd_3od) δ 7.49 (dd, J = 15.6, 3.2 Hz, 1H), 5.88 (dt, J = 14.9, 6.7 Hz, 1H), 5.87 – 5.82 (m, 1H), 5.25 (dd, J = 15.2, 9.3 Hz, 1H), 4.83 (td, J = 12.4, 6.3 Hz, 1H), 4.20 (d, J = 9.3 Hz, 1H), 2.83 – 2.75 (m, 1H), 2.71 (dd, J = 19.1, 8.5 Hz, 1H), 2.43 (dd, J = 18.7, 8.3 Hz, 1H), 2.22 (dd, J = 19.1, 10.5 Hz, 1H), 2.12 (dd, J = 18.7, 11.1 Hz, 1H), 2.03 (d, J = 9.4 Hz, 2H), 1.87 (ddd, J = 27.1, 14.4, 5.5 Hz, 2H), 1.80 – 1.70 (m, 1H), 1.65 – 1.54 (m, 1H), 1.23 (t, J = 11.1 Hz, 3H), 1.04 – 0.93 (m, 1H).

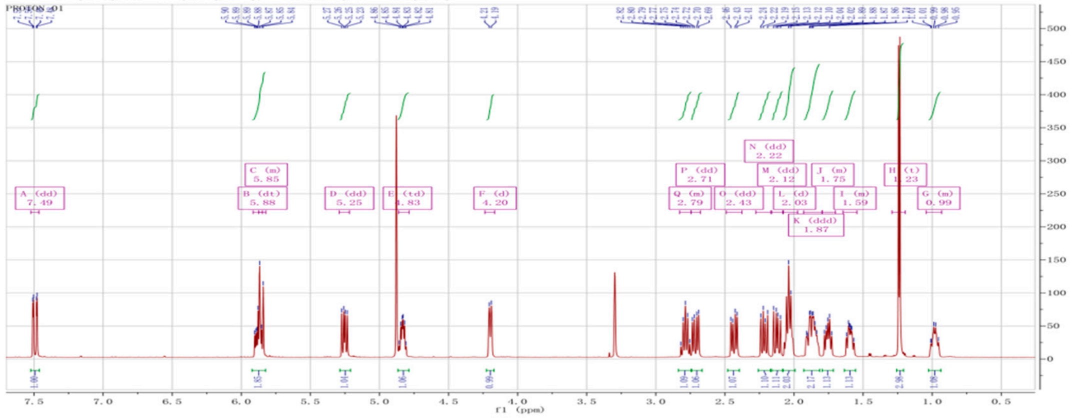

(b)

^{13}C NMR (151 MHz, cd_3od) δ 217.86, 166.78, 152.69, 135.74, 131.56, 116.86, 75.71, 71.65, 49.84, 45.89, 44.49, 42.08, 33.71, 31.24, 26.45, 19.56.

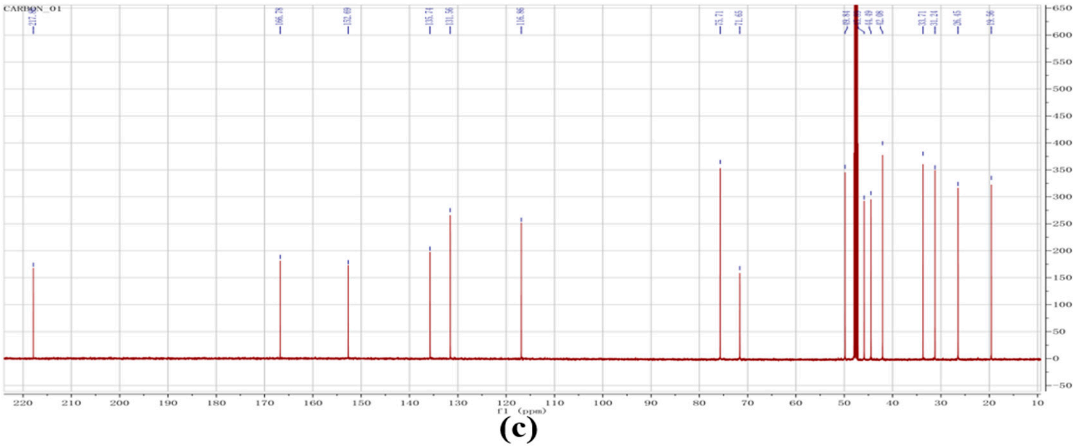

(c)

Figure S4: The MS and NMR spectra of 7-dehydrobrefeldin A (2): (a) MS spectrum, (b) ^{1}H NMR spectrum (CD_3OD), (c) ^{13}C NMR spectrum (CD_3OD).

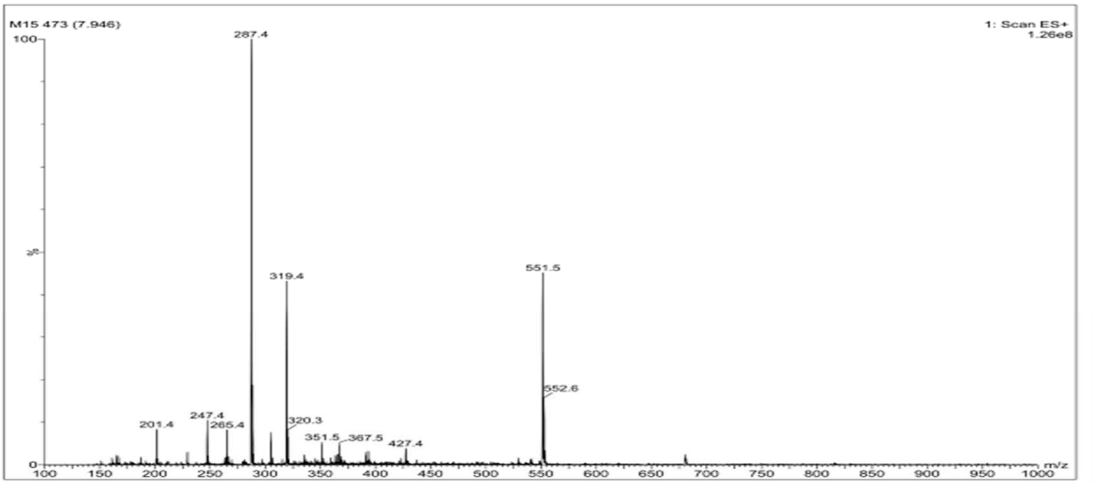

(a)

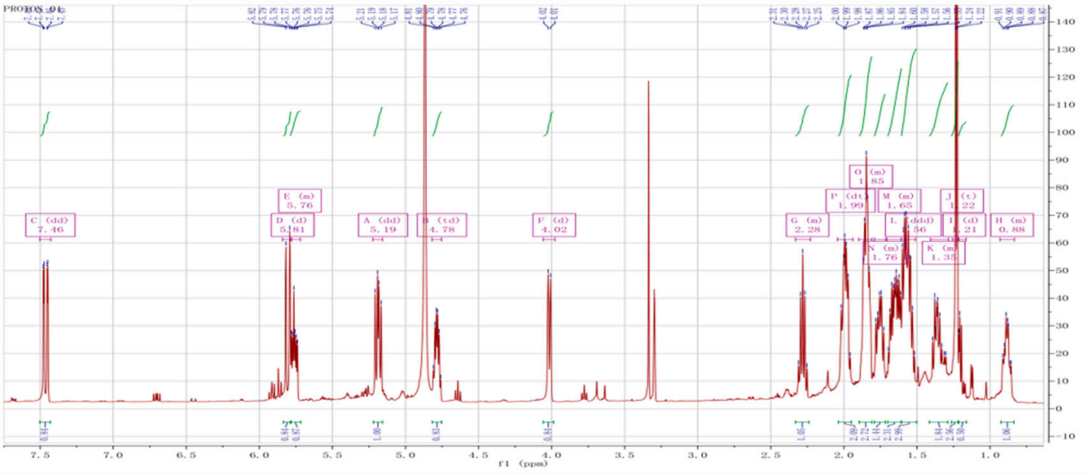

(b)

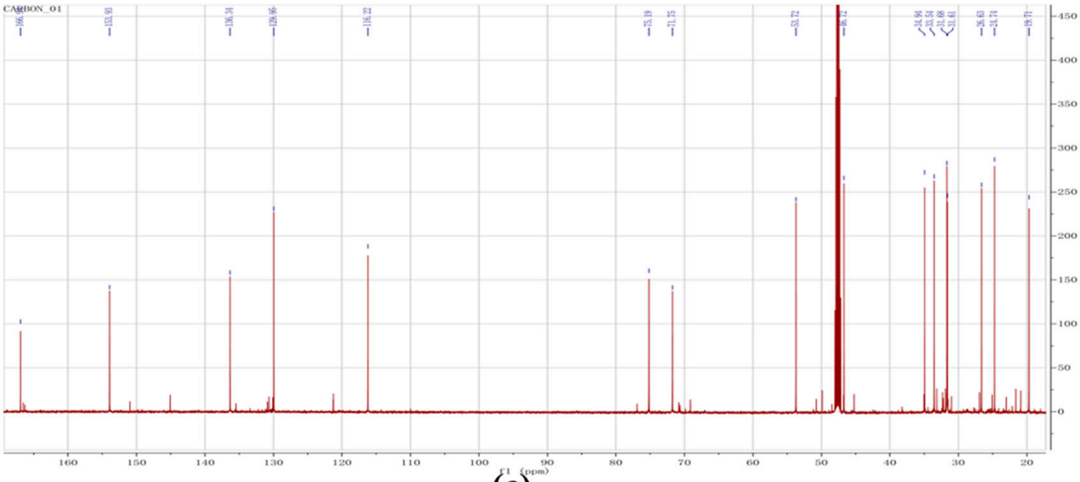

(c)

Figure S5: The MS and NMR spectra of brefeldin C (3): (a) MS spectrum, (b) ^{1}H NMR spectrum (CD_3OD), (c) ^{13}C NMR spectrum (CD_3OD).

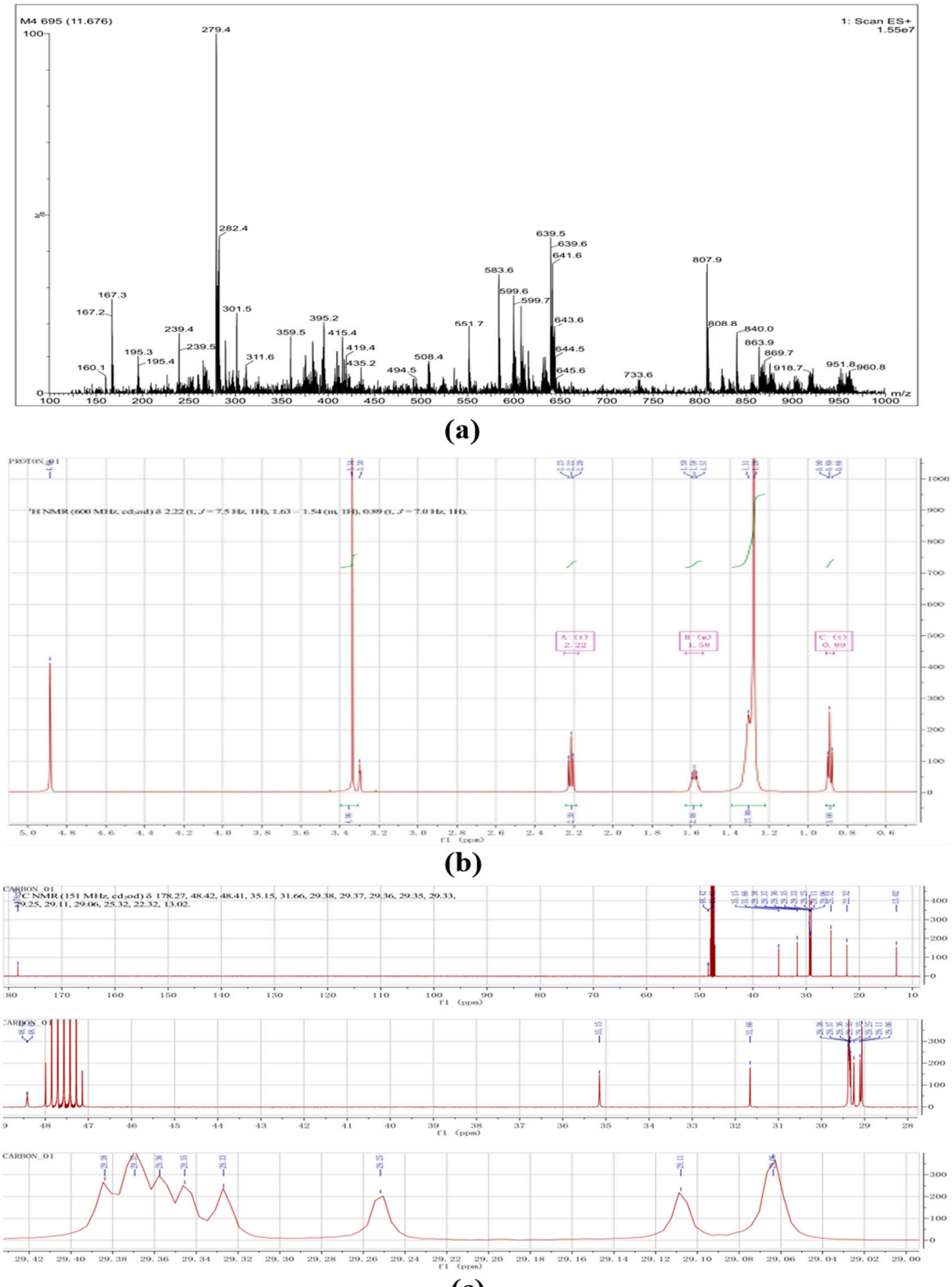

Figure S6: The MS and NMR spectra of methyl tetradecanoate (4): (a) MS spectrum, (b) ^{1}H NMR spectrum (CD_3OD), (c) ^{13}C NMR spectrum (CD_3OD).

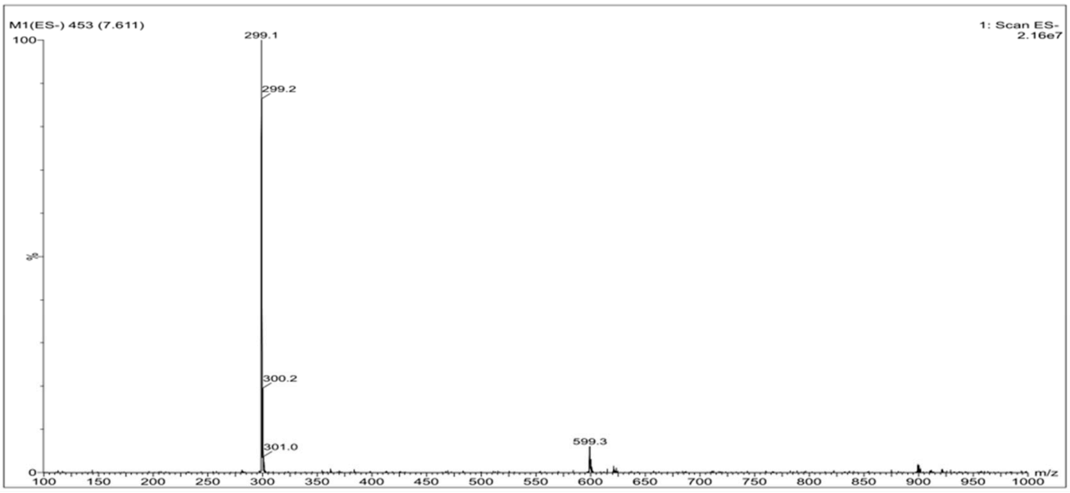

(a)

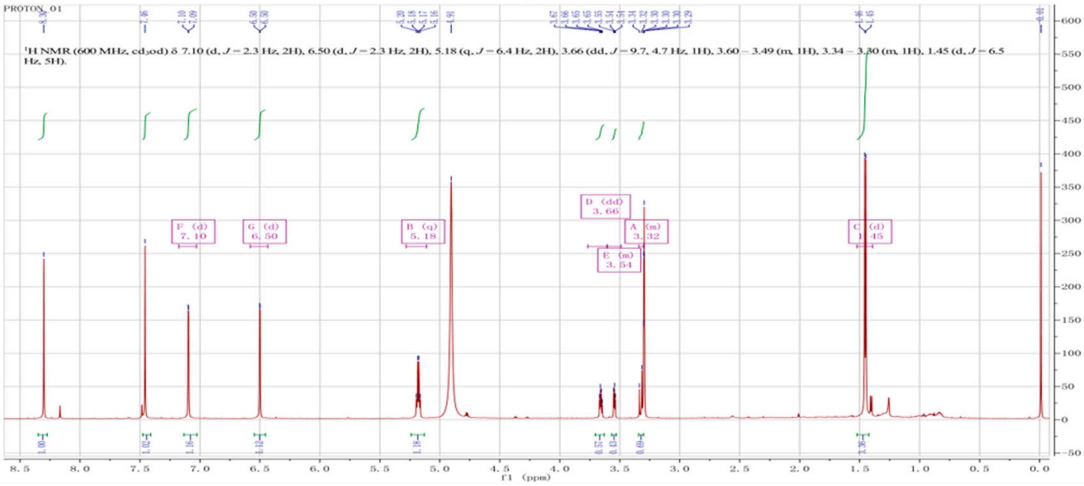

(b)

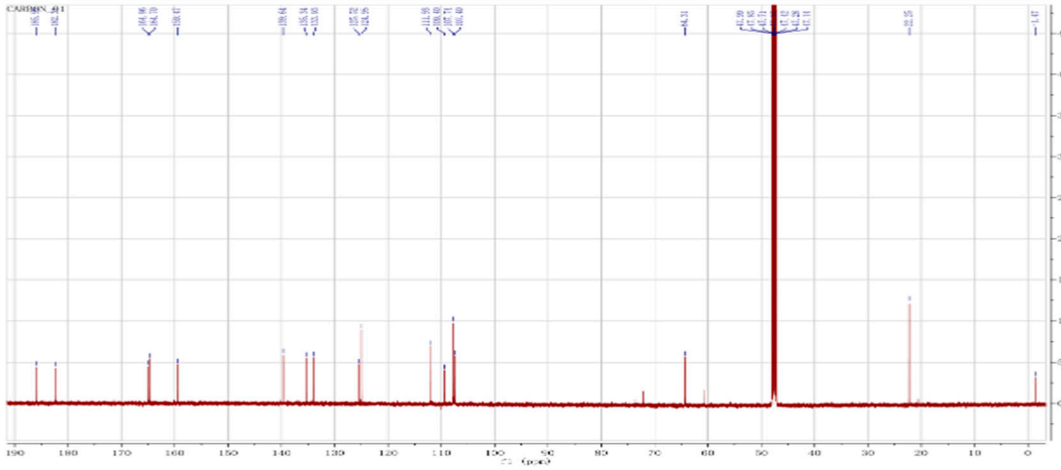

(c)

Figure S7: The MS and NMR spectra of anthraquinone ZSU-H85 (5): (a) MS spectrum, (b) ^{1}H NMR spectrum (CD_3OD), (c) ^{13}C NMR spectrum (CD_3OD).

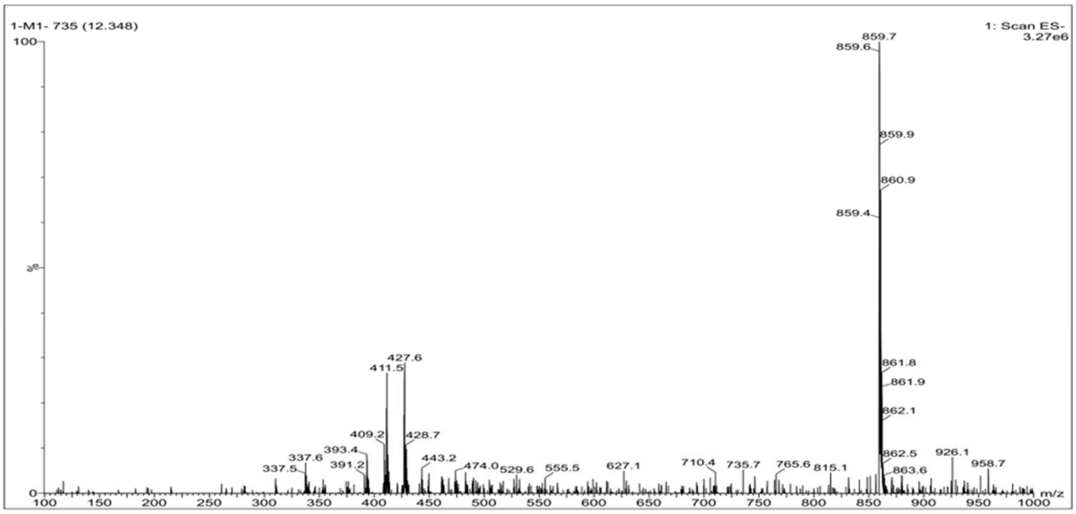

(a)

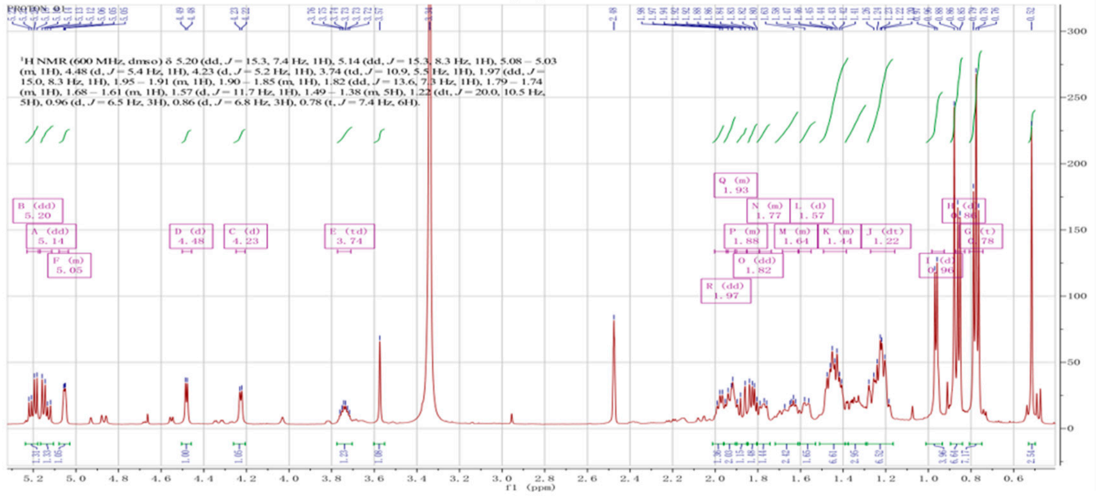

(b)

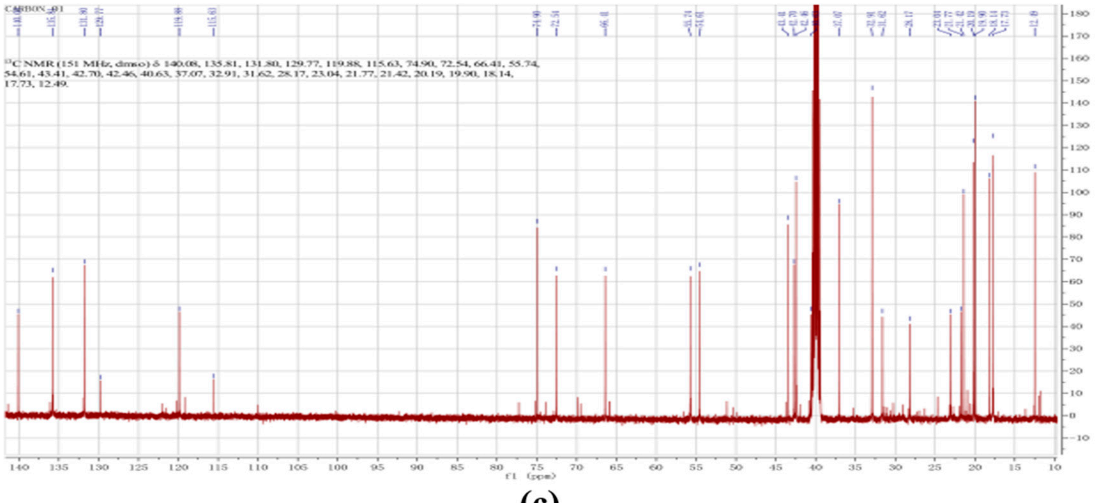

(c)

Figure S8: The MS and NMR spectra of (3β,5α,6β,22E)-ergosta-7,22-diene-3,5,6-triol (6): (a) MS spectrum, (b) ^{1}H NMR spectrum (DMSO-d_4), (c) ^{13}C NMR spectrum (DMSO-d_4).
